# Stereotactic radiosurgery and fractionated radiation therapy in the management of pituitary tumors

**DOI:** 10.1093/noajnl/vdae010

**Published:** 2024-12-19

**Authors:** Georgios Mantziaris, Daniel M Trifiletti, Stylianos Pikis, Jason P Sheehan

**Affiliations:** Department of Neurosurgery, University of Virginia, Charlottesville, Virginia, USA; Department of Radiation Oncology, Mayo Clinic, Jacksonville, Florida, USA; Department of Neurosurgery, University of Virginia, Charlottesville, Virginia, USA; Department of Neurosurgery, University of Virginia, Charlottesville, Virginia, USA

**Keywords:** pituitary, radiotherapy, stereotactic radiosurgery

## Abstract

Pituitary tumors require a diverse, multidisciplinary approach in their management. Stereotactic radiosurgery (single or hypofractionated) and fractionated radiotherapy are integral parts of the armamentarium. With the introduction of highly conformal, stereotactic ionizing radiation approaches, the reported tumor control rates and clinical outcomes have improved significantly. This review details the indications, risks, and outcomes of pituitary radiosurgery and radiation therapy, and presents in brief the differences in treatment approaches between stereotactic radiosurgery, fractionated radiation therapy, and proton therapy.

**Key Points**

- Radiotherapy techniques for pituitary neuroendocrine tumors include stereotactic radiosurgery, fractionated radiotherapy, and particle radiation.
- Stereotactic radiosurgery and fractionated radiotherapy offer high tumor and endocrine control.

According to the latest CBTRUS report, tumors of the pituitary gland represent the second most common reported intracranial histology comprising 17.1% of all primary central nervous system tumors.^1^ A significant increase in the diagnosis of pituitary tumors has occurred between 2004 and 2018; approximately 14,000 new cases were expected each year for 2021 and 2022 in the United States^1^ Despite recent advancements in surgical techniques and medical treatments, ionizing radiation remains an integral part of the management of functioning and nonfunctioning pituitary tumors. The primary aim for delivering radiation treatment to a pituitary tumor is the delivery of an effective treatment dose to the target tumor volume thereby affording cessation of tumor growth or regression and normalization of any hormonal overproduction while at the same time sparing surrounding normal tissues from any adverse radiation effects.

## Overview of Radiation-based Techniques

### Fractionated Radiation Therapy

Irrespective of the delivery method, standard fractionated radiation therapy (RT) is usually delivered in fractions of 1.8–2 Gy over more than 5 fractions to a total dose of 45–54 Gy for nonfunctioning pituitary adenomas and 50–55 Gy for functioning adenomas. Fractionated RT techniques have evolved from conformal 3-dimensional (C3D) radiotherapy to intensity-modulated radiotherapy (IMRT) and volumetric-modulated arc therapy (VMAT), a form of IMRT, to improve conformity with the shape of the tumor.^2^ The treatment is delivered using a linear accelerator (LINAC), typically with a noninvasive method of patient immobilization.

**Figure 1.**
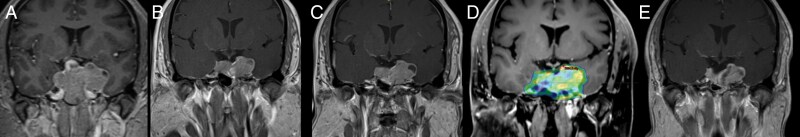
(A) A 47 year-old woman presented with peripheral visual field loss and was found to have a large nonfunctioning PitNET. (B) She underwent initial subtotal transsphenoidal resection, (C) and 1 year later she developed tumor progression. (D) She underwent salvage volumetric-modulated arc-based radiotherapy to 50.4 Gy in 28 fractions. (E) Four years post radiotherapy radiographic tumor control is maintained.

Currently, IMRT constitutes the standard approach in pituitary tumors; it utilizes lead leaves moving into the beam path (multi-leaf collimator—MLC) to vary radiation dose intensity across it. The treatment plan can be further customized with multiple beam arcs, such that the radiation dose can be delivered from different angles (VMAT). While these newer techniques do not significantly improve target volume dose coverage, they can improve conformity and allow for better preservation of organs at risk (OAR), such as the optic apparatus.^2,3^ With IMRT, the rates of tumor control and endocrine response remain high, while toxicity rates are low (Figure 1).^4–6^

### Stereotactic Radiosurgery

Stereotactic radiosurgery (SRS) delivers high doses of ionizing radiation, typically in a single treatment session, to a small target volume. Gamma Knife radiosurgery (Elekta AB, Stockholm) is 1 of the most common radiosurgery platforms for intracranial lesions; the current Gamma Knife Esprit is comprised of 192 ^60^Co sources that deliver beams of radiation converging on the target. A stereotactic frame is typically used for localization and correlation of target coordinates. However, following the introduction of the onboard cone-beam CT scan (CBCT) and infrared tracking with the Gamma Knife Icon model, mask-based localization has allowed for an easier fractionation of treatment schemes. By dividing treatment across up to 5 fractions, the treatment time for a single session can be reduced, and also larger tumors can be more safely treated.^7^

Common treatment platforms for linear accelerator (LINAC) SRS include the Cyberknife (Accuray Inc, Sunnyvale, USA), TrueBeam (Varian Inc, Palo Alto, USA), Versa HD (Elekta AB, Stockholm), and Zap (ZAP Surgical Systems Inc, San Carlos, California). A LINAC may use variable arrangements, such as noncoplanar arcs, dynamic rotation, and conical rotation. Additionally, MLCs allow for various combinations of the above and dynamic changes during beam motion. The radiation prescription dose is usually prescribed at higher isodose lines compared to the Gamma Knife, allowing for a more homogeneous distribution across the target.^8^

Radiosurgery planning typically involves MRI and CT-based planning. However, the use of PET imaging and DTI-based connectome mapping may help to further refine target volumes and nearby critical structures in treatment planning.^9^

### Particle Therapy

The majority of the experience regarding particle radiation therapy comes from the published series of proton beam radiotherapy (PBRT). The rationale behind proton beam radiation therapy is based on the theoretical advantage of maximum energy deposition at a defined depth in tissue with minimal effect after that point; this phenomenon (Bragg peak) enables the improved sparing of healthy tissues.^10^ However, this sharper dose fall-off sometimes necessitates more frequent image verification to assess for positional changes or rapid tumor response. Additionally, the treatment plan should take into account these uncertainties when defining the planning target volume.

Carbon ion radiotherapy (CIRT) is an emerging heavy particle treatment for skull base tumors. Due to their radiobiological properties, carbon ions show reduced scattering laterally and at depth compared to protons. The radiobiological properties of carbon ions expand the theoretical advantages of PBRT, providing an even sharper dose falloff and increasing its biological effectiveness.^11^ While currently there are no study results with PitNET patient outcomes, their effectiveness has been well demonstrated in other skull base tumors, such as chordomas and chondrosarcomas.^11^

Boron neutron capture therapy has shown promising results in the treatment of high-grade CNS tumors, as well as head and neck malignancies.^12^ However, its application on PitNET treatment is currently limited to in vitro studies.^13^

## Nonfunctioning Pituitary Neuroendocrine Tumors

Nonfunctioning (NF) pituitary neuroendocrine tumors (PitNET) are usually diagnosed due to mass effect symptoms; these might include cranial nerve deficits, headache owing to local mass effects, or hormonal dysfunction, such as hypopituitarism or stalk effect-associated hyperprolactinemia.^14^ The treatment algorithm usually involves maximal safe resection as primary management, either via a transsphenoidal or transcranial approach; SRS or radiation therapy is usually offered in the case of residual or progressive tumors.^15,16^

The available evidence is currently insufficient to support the superiority of single-fraction SRS (SF-SRS) over hypo-fractionated SRS (HF-SRS) and fractionated radiotherapy (RT). However, in clinical practice, single-fraction SRS represents a convenient option for patients with small tumors away from the optic pathway, while HF-SRS and RT are preferred in the case of larger lesions abutting the optic nerves/chiasm or in the cases of repeat irradiation.^17^

### Stereotactic Radiosurgery Outcomes

**Figure 2.**
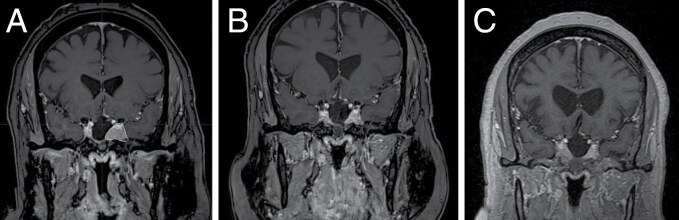
(A) Procedural, T1-weighted coronal image of a 64 year-old woman that presented with a progressive residual nonfunctioning PitNET involving the left cavernous sinus. She underwent single-session Gamma Knife radiosurgery, delivering a prescription dose of 15 Gy (yellow line) in a single fraction. (B) Radiographic tumor regression was noted at the 5-year follow-up. (B) Tumor control was maintained 10 years after the radiosurgical treatment.

Tumor control results of SRS for NF-PitNETs are mainly derived from retrospective studies; this level IV evidence was summarized in 2019 in the International Society of Stereotactic Radiosurgery (ISRS) meta-analysis and practice guidelines.^7^ Kotecha et al. included 35 studies and 2671 patients treated between 1971 and 2017; the 5- and 10-year tumor control were high at 94% and 83%, respectively^7^ (Figure 2).

The majority of patients included in the study by Kotecha et al. were treated using single-fraction SRS with a median peripheral dose of 15 Gy.^7^ Major retrospective single-fraction GKRS and LINAC studies are presented separately in Table 1.^18,19,21,25–27^ All studies report high crude (89.6–98.3%) and 5-year (94–95%) control rates, while 10-year rates are noticeably lower (76–85%).^18,19,21,25^ In a retrospective, single-center study by Losa et al. the majority of tumor recurrences were observed at a time interval between 5 and 10 years following treatment.^21^ While volumetric studies have shown a correlation between early tumor regression and sustained tumor control,^28^ as tumor control rates diminish with extended follow-up, it is paramount that longitudinal neuroimaging surveillance is maintained.

**Table 1.** Outcomes of Radiation Therapy for Nonfunctioning Pituitary Neuroendocrine Tumors.

Author, year	Patient number	Margin dose (Gy)	Median follow-up (months)	Tumor control	Hypopituitarism	Other deficits
GKRS series						
Park et al., 2011^18^	148	Median: 14 1fx	65	Crude: 86.5% 5-year: 88% 10-year: 74%	Crude: 27.5% 5-year: 30% 10-year: 50%	ON: 4.1% Other CN: 2.7%
Runge et al., 2012^19^	204	Median: 14 1fx	86	Crude: 90% 5-year: 95% 10-year: 92%	Crude: 18% 5-year: NR 10-year: NR	ON: 2.5% Other CN: 0 %
Sheehan et al., 2016^16^	272	Median: 15 1fx	79	Crude: 90.4% 5-year: 94.5% 10-year: 78.7%	NR	NR
Minniti et al., 2016^17^	512	Median: 16 1fx	36	Crude: 93.4% 5-year: 95% 10-year: 85%	Crude: 21.1% 5-year: NR 10-year: NR	ON: 6.6% Other CN: 2.7%
LINAC SRS/SBRT series						
Hamblin et al., 2022^20^	51	Median: 14 1fx	50	Crude: 100% 5-year: 100% 10-year: 100%	NR	ON: 2% Other CN: 0%
Losa et al., 2017^21^	61	Median: 13 1fx	83	Crude: 98.3% 5-year: NR 10-year: NR	Crude: 9.8% 5-year: 12.1% 10-year: 12.1%	ON: 0% Other CN: 0% Seizures: 1.7%
Verma et al., 2014^22^	100	17–21 Gy 3fx 22–25 Gy 5fx	33	Crude: 95% 5-year: 98% 10-year: NR	Crude: 4.1% 5-year: NR 10-year: NR	ON: 1% Other CN: 0%
FSRT/IMRT series						
Breen et al., 1998^23^	68	45 Gy 25fx	75	Crude: % 5-year: 97% 10-year: 91%	Crude: 26.5% 5-year: 40% 10-year: 72%	ON: 2.9% Other CN: 0%
Hamblin et al., 2022^20^	67	14.4–53.6 Gy 5–30fx	61	Crude: 91% 5-year: 92.8% 10-year: 85.7%	NR	ON: 2% Other CN: 0%
PBRT series						
Brada et al., 2002^24^	24	44–53.3 Gy 28–30fx	47	Crude: 100% 5-year: 100% 10-year: 100%	Crude: 20% 5-year: NR 10-year: NR	ON: NR Other CN: NR Temporal lobe necrosis: 4.3%

GKRS: Gamma Knife radiosurgery, LINAC: Linear accelarator, FSRT: Fractionated stereotactic radiotherapy, IMRT: Intensity-modulated radiotherapy, PBRT: Proton beam radiotherapy.

New-onset hypopituitarism is the most common side effect, with the reported pooled estimate being 21%.^7^ Hypopituitarism occurs most commonly within 5 years after radiosurgery, but there is a risk of delayed hypopituitarism even 10 years after radiosurgery.^29^ A variety of dosimetric parameters have been proposed as predictors of future pituitary dysfunction, including prescription isodose line, mean pituitary dose, and the maximum dose delivered to the pituitary stalk. Specific thresholds, such as a maximum dose of 8–10 Gy on the pituitary stalk, have been verified as independent predictors in multiple studies.^29–31^

Cranial nerve dysfunction, carotid injury and subsequent stroke, and radiation-induced tumors are rarely seen following SRS.^7,32^ However, attention is required with tumors that are nearby (<3 mm) to the optic pathways. Regarding the optimal prescription dose for SF-SRS, some authors have suggested that thresholds as low as 12 Gy might be sufficient to achieve tumor control; it should be noted though that these studies usually include very few patients.^33^ The current ISRS practice guidelines recommend 14–16 Gy in a single fraction.^7^ The patient’s eligibility for SF-SRS, as well as the prescribed dose are heavily dependent on the proximity of the tumor to the optic pathway; as such, unless the optic apparatus tolerance limits (<10 Gy in 1 fraction) are exceeded,^34^ the prescribed dose could be escalated. It has been suggested, that patients with more aggressive subtypes (such as silent corticotroph PitNET) might benefit from higher radiation doses, though some study reports are contradictory.^35,36^

The evidence regarding the efficacy of HF-SRS for NF-PitNETs, especially long-term outcomes on tumor control, is more limited. In the meta-analysis by Kotecha et al. that included 220 patients treated with various hypo-fractionated schedules (21 Gy in 3 fractions, 20 Gy in 4 fractions, and 25 Gy in 5 fractions), the 5-year pooled tumor control estimate of HF-SRS studies was comparable to SF-SRS studies at 97%; the lack of long-term data prevented the authors from providing the equivalent 10-year control rate.^7^ Using the linear quadratic model and assuming an *α*/*β* ratio of 3 Gy, these schedules appear to be biologically equivalent to 11–13 Gy in a single fraction.^7^ Due to the limited long-term results and the heterogeneity of fractionation schedules, such regimens could be considered for patients with tumors in close proximity to the optic pathways or a history of sella irradiation.

### Fractionated Radiotherapy Outcomes

A number of historic large cohorts provide data on the long-term effectiveness of conventional RT in maintaining tumor control and associated toxicity. The actuarial control rates are between 87% and 97% at 10 years and 92% at 20 years.^23,37,38^ Hypopituitarism is the most common long-term complication following conventional RT; in the largest series to date, Brada et al. reported a 30% probability of new hypopituitarism 10 years after receiving RT.^37^ The incidence of new visual deficits is approximately 1.5%.^37^ While optic pathway toxicity is relatively low, adults receiving conventional sella irradiation for PitNETs are at increased risk of radiation-induced brain tumors.^20^ Additionally, an increased risk of cerebrovascular mortality has been described in patients treated with RT relative to the general population, although it is not clear the extent to which RT contributes to this excess risk.^24^ Owing to these late-term risks, preference has shifted toward more conformal techniques, such as SRS and fractionated stereotactic radiotherapy (FSRT) or IMRT with doses between 45 and 50.4 Gy in 25–28 fractions provides tumor control rates similar to those achieved by SRS and conventional RT (Table 1). SRS could in theory partially mitigate the risk of vasculopathy and stroke, as compared with fractionated RT, due to the tight radiation margins and sharp dose fall-off; however, no definitive studies evaluating the cerebrovascular adverse effects of SRS have been performed to date. Due to the radiobiological advantages of dose fractionation in reducing the risk of radiation-induced toxicity, it is well-suited for patients with large tumors in close proximity to critical neurovascular structures. Minniti et al. in their series of 68 patients with large, invasive NF-PitNETs reported favorable long-term tumor control (10-year 91%).^39^ The reported crude rate of hypopituitarism is 26.5%, but, as FSRT generally does not avoid the pituitary stalk and hypothalamus, this rate could approach the ones reported in conventional RT with extended follow-up.^39^ Other late complications, such as RION, radiation-induced neoplasia, and cranial nerve dysfunction, appear to be low.^39,40^

### Proton Beam Radiotherapy Outcomes

The available published reports on PBRT for NF-PitNETs do not currently provide sufficient evidence to support the theoretical advantage of PBRT over photon-based modalities. In the most recent study by Ronson et al. that included 47 patients (24 patients with NF-PitNETs), 5,040–5,590 cobalt-gray equivalent were delivered to the gross tumor volume over 28-30 fractions. At a median follow-up time of 47 months, the authors reported 100% radiographic tumor control. Regarding morbidity in this series (including the functioning PitNETs), 1 patient developed temporal lobe necrosis, 3 developed new significant visual deficits, and 11 developed new hypopituitarism.^41^

### Treatment Timing

There is currently no consensus on the optimal time of radiation treatment after surgical resection of NF-PitNETs.^16^ Regarding SRS, there are conflicting reports on whether early treatment (within 6 months following resection) yields improved clinical and radiographic outcomes, relative to treatment at the time of radiographic tumor progression.^29,42^ While this threshold of asymptomatic, radiographic tumor progression constitutes a reproducible benchmark for further study, it can potentially lead to the development of symptoms or narrow therapeutic options. On the other hand, early treatment may accelerate the development of side effects, such as hypopituitarism, thus causing unnecessary treatment-related morbidity. As such, the risk of clinical deterioration due to tumor progression should be weighed against the risk of radiation-induced morbidity on a per-patient basis. Also, the duration of the risk period and preferences of patients should be considered in deciding on early treatment versus active surveillance of a residual NF-PitNET.

### Repeat Irradiation

Late recurrences after radiotherapy, while rare due to the high rates of local tumor control, have been reported, and as such long-term neuroimaging follow-up is recommended. Together with resection, repeat irradiation (either with SRS or FSRT) should be carefully considered, as it is associated with an increased risk of radiation-induced damage to the optic pathways, cranial nerves, and surrounding brain parenchyma. Small retrospective series have demonstrated the feasibility of reirradiation for patients with disease recurrence after previous pituitary radiotherapy.^22,43^ In principle, the degree of overlap between the treatment fields, the time interval between radiotherapy treatments, and the type of radiation therapy can greatly influence the rate of toxicity. Two small studies, with each reporting outcomes on 15 patients with various NF- and functioning (F-) PitNETs, focus exclusively on pituitary repeat irradiation; toxicity rates were high and included hypopituitarism, radiation-induced optic neuropathy (RION), and temporal lobe necrosis.^22,43^

## Functioning Pituitary Neuroendocrine Tumors

Functioning PitNETs (F-PitNET) include a range of tumors with variable histopathology and clinical characteristics; their common attribute is the hypersecretion of pituitary hormones. While outcomes are usually reported separately, the principles behind the SRS or RT management generally remain the same. Similar to NF-PitNETs, recommendations are mainly derived from retrospective studies. SRS or radiation therapy is usually reserved as a third-line option, usually in the setting of hormonal hypersecretion that remains uncontrolled after medical treatment and surgical resection.

### Cushing’s Disease

Cushing’s disease arises due to adrenocorticotropin (ACTH)-secreting PitNETs, resulting in high cortisol levels.^44^ Transsphenoidal resection (TSR) is usually the first-line treatment approach, and it leads to remission in 60–80% of the patients.^44^ SRS or radiation therapy is employed as an adjuvant in cases of recurring or persistent disease following TSR.^44^ Retrospective studies have demonstrated the effectiveness of both fractionated RT and stereotactic radiosurgery in Cushing’s disease^45–49^ (Table 2).

**Table 2.** Outcomes of Radiation Therapy for ACTH-secreting Pituitary Neuroendocrine Tumors.

Author, year	Patient number	Radiation dose (Gy)	Follow-up (months)	Sustained endocrine control	Toxicity
Radiosurgery series					
Schoenthaler et al., 1992^43^	278	Mean: 23.7	Mean: 67	Crude: 57% 5-year: 62% 10-year: 64% Recurrence: 18%	Hypopituitarism: 25% ON: 1% Other CN: 1%
Fleseriu et al., 2021^44^	35	Mean: 14.7	Mean: 42	Crude: 49% 5-year: NR 10-year: NR Recurrence: NR	Hypopituitarism: 40% ON: NR Other CN: NR
Fractionated RT series					
Mehta et al., 2017^45^	40	45–50 Gy	Median: 108	Crude: 83% 5-year: 78% 10-year: 84% Recurrence: 0%	Hypopituitarism: 73% ON: 2.5% Other CN: 0%
Devin et al., 2004^46^	30	48–54 Gy	Median: 42	Crude: 83% 5-year: 91.7% 10-year: NR Recurrence: 0%	Hypopituitarism: 57% ON: 0% Other CN: 0%
PBRT series					
Petit et al., 2008^49^	79	Median: 20 Gy or 50.4–54 Gy 28–30 fx	Median: 47	Crude: 64% 5-year: 67% 10-year: NR Recurrence: 1%	Hypopituitarism: 57% ON: 0% Other CN: 2.5% Seizures: 3.8%
Minniti et al., 2007^47^	33	Median: 20 Gy	Median: 62	Crude: 52% 5-year: 55% 10-year: NR Recurrence: 0%	Hypopituitarism: 42% ON: 0% Other CN: 0%

Fractionated RT for Cushing’s disease results are derived mainly from small retrospective series; sustained, long-term remission, with reported rates approximating 83%, have been reported.^47,48^ The mean time to remission is 18–24 months, and most series do not report endocrine recurrences.^47,48^ Radiographic tumor control rates are also high, with more than 90% demonstrating stability or regression at the last follow-up.^47^ Typically, 45–54 Gy are delivered over multiple days of treatment in daily fractions of 1.8–2 Gy. The main concern with fractionated RT is the exceedingly high rates of new pituitary dysfunction; 57–73% of treated patients will develop a dysfunction of at least 1 pituitary axis during their follow-up.^47,48^

On the contrary, sustained remission rates achieved with SRS appear to be lower; in the largest retrospective SRS study with 278 patients, initial biochemical control was achieved in 80% with a median time to normalization of 12 months but durable remission was maintained in 57% of the patients.^45^ However, new pituitary endocrinopathy was also markedly lower at 25% at the last follow-up.^45^ Whether the improved targeting of SRS, which might help spare normal pituitary function, is also responsible for the under-treatment of microscopic seeding and thus higher recurrence, is a matter of debate. Also, SRS appears to induce faster remission than RT.^50^

Proton RT, either fractionated or single-session, appears to offer remission rates comparable to photon-based SRS; the evidence behind the efficacy of this radiation treatment though is limited to a few, small single-center studies.^49,51^ New pituitary deficiency was slightly more common than with photon SRS; temporal lobe radiographic changes, occasionally presenting with seizures, have also been reported.^49,51^

The variability in reported outcomes could be attributed to the differences in cohort size, remission criteria employed, and duration of follow-up. Despite that, there is sufficient evidence to suggest that radiation therapy, either fractionated RT or SRS, affords reasonable rates of disease remission. SRS is probably more convenient but is subject to OAR tolerance limits. Lifelong clinical, radiographic and laboratory monitoring for new hormone deficiencies, disease recurrence, and radiation adverse effects is required.^44^

### Nelson’s Syndrome

Nelson’s syndrome is characterized by the accelerated growth of an ACTH-secreting PitNET and excessive secretion of ACTH following bilateral adrenalectomy (BA).^52^ In a recent systematic review and meta-analysis of 37 studies and 1316 patients treated with BA, the pooled prevalence of Nelson’s syndrome was 26%.^52^ TSR generally remains the preferred approach when feasible, affording high remission rates (70–80%) in selected cases.^53^

Pituitary RT has been used both prophylactically to prevent the development of Nelson’s syndrome and as a secondary treatment option to control tumor progression and ACTH secretion.^53^ In the former case, in a retrospective series of 43 patients treated with BA and prophylactic SRS (33 with SRS before BA and 10 without), RT significantly reduced the risk of Nelson’s syndrome development.^54^ However, due to the conflicting reports regarding its benefits, routine prophylactic pituitary irradiation is not recommended.^53^

Concerning the outcomes of RT in patients with diagnosed Nelson’s syndrome, they are in general less favorable when compared to other forms of F-PitNETs. While earlier fractionated RT series provided poorly documented outcome data, recent advances may limit the translatability to modern radiotherapy.^53^ The majority of recent studies report outcomes following Gamma Knife SRS; at last follow-up tumor control rate can exceed 90%, while ACTH level reduction can be achieved by two-thirds of the patients.^31^

### Acromegaly

Acromegaly arises due to growth hormone (GH)-secreting PitNETs, resulting in high IGF-1 levels.^55^ TSR is the first-line treatment approach, and resection results in an initial remission rate between 40% and 85%, depending on tumor size. SRS or RT is usually recommended as an adjuvant in cases of uncontrolled GH secretion after surgery and medical therapy.^55^

The long-term efficacy and adverse effects of fractionated RT have been well-documented in multiple studies^4,56,57^ (Table 3). Results from the UK National Acromegaly Register Study Group presented the long-term results of the largest cohort to date; of 886 acromegalic patients, the 10-year remission rate was 60% at the cost of an equally high rate of hypopituitarism.^56^ The development of more conformal techniques have helped to mitigate the risk of delayed pituitary dysfunction, while still maintaining adequate endocrine control.^4,57^

**Table 3.** Outcomes of Radiation Therapy for GH-secreting Pituitary Neuroendocrine Tumors.

Author, year	Patient number	Radiation dose (Gy) )	Follow-up (months)	Sustained endocrine control without medication	Toxicity
Radiosurgery series					
Knappe et al., 2020^57^	371	Mean: 24.2	Mean: 78.9	Crude: 45% 5-year: 43% 10-year: 59% Recurrence: 9%	Hypopituitarism: 26.1% ON: 3.5% Other CN: 0.8%
Jenkins et al., 2006^56^	119	NR	Mean: 107	Crude: 37% 5-year: NR 10-year: 48% Recurrence: NR	Hypopituitarism: NR CN palsy: NR ON: NR
Ding et al., 2019^58^	138	Median: 25	Mean: 82.5	Crude: 34.1% 5-year: 20.3% 10-year: 44.9% Recurrence: NR	Hypopituitarism: 8.6% ON: 0% Other CN: 0%
Fractionated RT series					
Katznelson et al., 2014^55^	884	Median: 45 Gy 25fx	Median: 84	Crude: 5-year: 36% 10-year: 60% Recurrence: NR	Hypopituitarism: 58% ON: 0% Other CN: NR
Lian et al., 2020^4^	113	50–56 Gy 25–30 fx	Median: 36	Crude: NR 5-year: 69.9% 10-year: NR Recurrence: 2.7%	Hypopituitarism: 28.3% ON: 0% Other CN: 0%
Jenkins et al., 2006^56^	233	NR	Mean: 156	Crude: 33% 5-year: NR 10-year: 52% Recurrence: NR	Hypopituitarism: NR ON: NR Other CN: NR
Proton beam RT series					
Petit et al., 2008^49^	61	Median: 20 Gy or 50.4–54 Gy 28–30 fx	Median: 57	Crude: 64% 5-year: 49% 10-year: NR Recurrence: 0%	Hypopituitarism: 62% ON: 0% Other CN: 0%

The reported rates of remission following SRS also vary in the published literature.^57–59^ The most recent systematic review and practice recommendations by the ISRS reported random effect estimates of 44% for crude endocrine remission and 17% for new-onset pituitary function loss.^60^ In the largest retrospective SRS series to date, Ding et al. reported that the 10-year sustained endocrine remission without medication was 59%.^58^ However, the definitions of remission used across the different studies are variable, with few reporting outcomes in accordance with the acromegaly consensus criteria.^61^

### Prolactin-secreting PitNET

PRL-secreting PitNET accounts for approximately 40% of all pituitary tumors; the resulting hyperprolactinemia may cause infertility, galactorrhea, neurological dysfunction due to mass effect or it may remain asymptomatic.^62^ Most patients with PRL-secreting PitNETs achieve biochemical control and radiographic tumor regression with pharmacologic treatment (dopamine agonists); TSR represents the next best option for patients intolerant or unresponsive to medical treatment, while RT is reserved for resistant or malignant tumors.^62^

Endocrine remission rates vary across RT methods and studies (Table 4). Three large SRS series (>100 patients each) provide most of the evidence for RT in prolactinomas; crude endocrine remission rates ranged between 23% and 43% and post-SRS new pituitary dysfunction occurred in approximately 25% of the patients.^63–65^ The latency period for endocrine control is typically on the order of 2–5 years.^63–65^ The evidence behind fractionated RT and proton RT is fairly limited, but the results appear to be comparable to the ones provided by photon SRS series.^51,66^

**Table 4.** Outcomes of Radiation Therapy for PRL-secreting Pituitary Neuroendocrine Tumors.

Author, year	Patient number	Radiation dose (Gy)	Follow-up (months)	Sustained endocrine control without medication	Toxicity
Radiosurgery series					
Melmed et al., 2011^62^	289	Median: 22	Median: 60	Crude: 43% 5-year: 41% 10-year: NR Recurrence: 5%	Hypopituitarism: 25% ON: 3% Other CN: NR
Hung et al., 2019^63^	176	Mean: 22.4	Median: 67	Crude: 23.3% 5-year: NR 10-year: NR Recurrence: NR	Hypopituitarism: 26% ON: 0% Other CN: 0%
Wan et al., 2009^64^	128	Mean: 31.5	Mean: 33.2	Crude: 28% 5-year: NR 10-year: NR Recurrence: 4.7%	Hypopituitarism: NR ON: 0% Other CN: 0%
Fractionated RT series					
Pan et al., 2000^65^	64	40–52 Gy 20–25 fx	Median: 88	Crude: 39% 5-year: 10% 10-year: 25% Recurrence: NR	Hypopituitarism: 35% ON: NR Other CN: NR
Proton beam RT series					
Petit et al., 2008^49^	9	Median: 20 Gy or 50.4–54 Gy 28–30 fx	Median: 71	Crude: 57% 5-year: 38% 10-year: NR Recurrence: 0%	Hypopituitarism: 35% ON: 0% Other CN: 0%

### Tumor Control Overall for F-PitNETs

Radiation therapy affords excellent tumor control rates, usually higher than 90% for all F-PitNETs.^60^ However, differentiating between tumor stabilization and tumor regression and aggregating the results among studies is challenging. Similar to NF-PitNETs, the variability in outcome definitions and methods of presentation are partly responsible. Regardless, the rate of tumor shrinkage continues to increase over time.

### Anti-secretory Medication and Timing of SRS

A major issue of controversy regarding RT is whether the discontinuation of anti-secretory medication during RT improves the rates of endocrine remission. Most of the evidence supporting this corroboration comes from retrospective SRS series. Regarding acromegaly, cessation of long-acting somatostatin analogs and dopamine agonists before and at the time of SRS was associated with higher rates of both initial [hazard ratio (HR) = 2.73, 95% CI: 1.41–5.31] and durable (HR = 2.49, 95% CI: 1.21–5.11) remission.^58^ Similar findings have been reported for ketoconazole in Cushing’s disease and dopamine agonists in the setting of PRL-secreting PitNETs.^67,68^ While these findings have not been consistently replicated across all studies, clinical practice has generally shifted toward temporarily withholding the respective medication at the time of SRS.^44,58,68^ It should be noted that if temporary medication cessation is not feasible for a patient, this should not preclude radiation treatment.

### Dose and Fractionation for F-PitNET

Concerning tumor control, it is considered that single-session prescription doses ranging between 12 and 20 Gy are sufficient to achieve durable radiographic tumor control; these recommendations are mainly derived from NF-PitNET studies.^60^ However, it is believed that higher doses are required to stop the cellular mechanisms behind hormone secretion and achieve endocrine remission.^60^ While there is currently no clear dose threshold that is required, dose escalation (>20 Gy) can be considered when dosimetric constraints of nearby OARs can be met.

The overwhelming majority of studies have reported outcomes using single-fraction SRS. Two small studies have reported promising outcomes on acromegalic patients treated with hypo-fractionated SRS.^69,70^ Hypo-fractionated SRS may be a reasonable option for patients with tumors near the optic pathways, but the advantages in terms of tumor control, endocrine remission, and toxicity rates compared to single-session SRS remain to be proved. For invasive, large tumors with anatomical or toxicity-related concerns fractionated RT might be the preferred approach (Table 5).

**Table 5.** Conclusions and Recommendations for Radiation Therapy of PitNEts.^7,31,59^

Summary for nonfunctioning PitNETs		
	Recommendation or conclusion	
Treatment timing	Tumor recurrence/progression Early salvage can be considered	
Dose and fractionation	SRS	• SF-SRS is preferred to HF-SRS if constraints to OARs can be met • For SF-SRS 14–16 Gy is recommended • HF-SRS can be considered for patients with larger adenomas (>2–3 cm) or close to the optic pathway • For HF-SRS 21 Gy in 3 fractions, 20 Gy in 4 fractions, or 25 Gy in 5 fractions is recommended
	Fractionated RT	• 45–50.4 Gy at 1.8–2 Gy per fraction • 25–28 fx
Tumor control	SRS	5-year • 94% for SF-SRS • 97% for HF-SRS 10-year • 83% for SF-SRS • Lacking data for HF-SRS
	Fractionated RT	10-year: >90% 20-year: >80%
Hypopituitarism	SRS	• 21% after SF-SRS • 3% after HF-SRS (limited data)
	Fractionated RT	10-year: 30–60%
Other toxicity	SRS	• RION risk < 1% with maximum point dose to optic apparatus of <10 Gy in 1 fx, <20 Gy in 3 fx and <25 Gy in 5 fx (in patients without prior RT) • >10-fold increase in crude rate with prior RT
	Fractionated RT	• <54 Gy to the optic pathway • 1–3% RION • Increased risk of stroke and radiation-induced tumors

Summary for functioning PitNETs		
	Recommendation or conclusion	
Treatment timing	• Medical management and resection are usually the first treatment options • Radiotherapy reserved for recurrent or resistant tumors and malignant disease	
Tumor control	• >90% overall • Tumor regression rises progressively over 2–3 years	
Dose and fractionation	SRS	• For SF-SRS doses 20–25 Gy might increase chance of endocrine remission • Increase prescription dose based on OAR dosimetric constraints • HF-SRS can be considered for patients with larger adenomas (>2–3 cm) or close to the optic pathway • Lacking data to recommend dose for HF-SRS
	Fractionated RT	• 50.4–54 Gy at 1.8–2 Gy per fraction • 25–30fx
Endocrine remission	SRS	• Cushing’s disease: 48% • Acromegaly: 44% • Prolactinomas: 28%
	Fractionated RT	• Cushing’s disease: >80% 10-year • Acromegaly: 50–60% 10-year • Prolactinomas: 25% 10-year
Hypopituitarism	SRS	12–21% after SF-SRS
	Fractionated RT	28–73%
Other toxicity	SRS	• RION risk <1% with maximum point dose to optic apparatus of <10 Gy in 1 fx, <20 Gy in 3 fx and <25 Gy in 5 fx (in patients without prior RT) • >10-fold increase in crude rate with prior RT
	Fractionated RT	• <54 Gy to the optic pathway • 1–3% RION • Increased risk of stroke and radiation-induced tumors
Anti-secretory medication	Withdrawal of anti-secretory medications preferred in SRS setting (4–12 weeks prior)	

## Summary of Conclusions and Recommendations

### Future Directions

Technological improvements in radiotherapy delivery technologies have allowed the development of new modalities, such as conformal RT, SRS, and proton beam therapy, which offer improved dose distributions. The introduction of these conformal techniques has significantly reduced toxicity while keeping tumor control rates high. New radiation techniques, such as heavy particle therapy, advances in PET and DTI-based imaging, could potentially optimize treatment and help lower the rate of side effects.

## Notes

Funding

This article appears as part of the supplement “Pituitary Tumors,” sponsored by Toronto Western Hospital, Neurosurgery Department.

Conflict of interest statement

The authors have no relevant financial or nonfinancial interests to disclose.

## References

[1] Ostrom QT , Cioffi G, Waite K, Kruchko C, Barnholtz-Sloan JS. CBTRUS statistical report: primary brain and other central nervous system tumors diagnosed in the United States in 2014–2018. Neuro Oncol. 2021;23(Suppl 3):iii1–iii105. 34608945 10.1093/neuonc/noab200 PMC8491279

[2] Ajithkumar T , Brada M. Stereotactic linear accelerator radiotherapy for pituitary tumors. Treat Endocrinol. 2004;3(4):211–216. 16026103 10.2165/00024677-200403040-00002

[3] Kosmin M , Fersht N. Radiotherapy for pituitary tumors. In: Feingold KR, Anawalt B, Blackman MR, et al, eds. Endotext. MDText.com, Inc.; 2000. Accessed June 2, 2023. http://www.ncbi.nlm.nih.gov/books/NBK278955/

[4] Lian X , Shen J, Gu Z, et al Intensity-modulated radiotherapy for pituitary somatotroph adenomas. J Clin Endocrinol Metab. 2020;105(12):dgaa651. 32930785 10.1210/clinem/dgaa651

[5] Mackley HB , Reddy CA, Lee SY, et al Intensity-modulated radiotherapy for pituitary adenomas: the preliminary report of the Cleveland clinic experience. Int J Radiat Oncol Biol Phys. 2007;67(1):232–239. 17084541 10.1016/j.ijrobp.2006.08.039

[6] Ramos-Prudencio R , Pérez-Álvarez SI, Flores-Balcazar CH, et al Radiotherapy for the treatment of pituitary adenomas: a dosimetric comparison of three planning techniques. Rep Pract Oncol Radiother. 2020;25(4):586–593. 32508534 10.1016/j.rpor.2020.04.020 PMC7264003

[7] Kotecha R , Sahgal A, Rubens M, et al Stereotactic radiosurgery for non-functioning pituitary adenomas: meta-analysis and International Stereotactic Radiosurgery Society practice opinion. Neuro Oncol. 2020;22(3):318–332. 31790121 10.1093/neuonc/noz225 PMC7058447

[8] Stieber VW , Bourland JD, Tome WA, Mehta MP. Gentlemen (and ladies), choose your weapons: Gamma Knife vs. linear accelerator radiosurgery. Technol Cancer Res Treat. 2003;2(2):79–86. 12680787 10.1177/153303460300200202

[9] Dayawansa S , Schlesinger D, Mantziaris G, et al Incorporation of brain connectomics for stereotactic radiosurgery treatment planning. Oper Neurosurg (Hagerstown). 2023;25:1. 37543746 10.1227/ons.0000000000000818

[10] Jette D , Chen W. Creating a spread-out Bragg peak in proton beams. Phys Med Biol. 2011;56(11):N131–N138. 21558588 10.1088/0031-9155/56/11/N01

[11] Lu VM , O’Connor KP, Mahajan A, Carlson ML, Van Gompel JJ. Carbon ion radiotherapy for skull base chordomas and chondrosarcomas: a systematic review and meta-analysis of local control, survival, and toxicity outcomes. J Neurooncol. 2020;147(3):503–513. 32206977 10.1007/s11060-020-03464-1

[12] Malouff TD , Seneviratne DS, Ebner DK, et al Boron neutron capture therapy: a review of clinical applications. Front Oncol. 2021;11:601820. 33718149 10.3389/fonc.2021.601820 PMC7952987

[13] Dai C , Cai F, Hwang KC, et al Folate receptor-mediated boron-10 containing carbon nanoparticles as potential delivery vehicles for boron neutron capture therapy of nonfunctional pituitary adenomas. Sci China Life Sci. 2013;56(2):163–173. 23334699 10.1007/s11427-012-4433-5

[14] Ntali G , Wass JA. Epidemiology, clinical presentation and diagnosis of non-functioning pituitary adenomas. Pituitary. 2018;21(2):111–118. 29368293 10.1007/s11102-018-0869-3

[15] Lucas JW , Bodach ME, Tumialan LM, et al Congress of neurological surgeons systematic review and evidence-based guideline on primary management of patients with nonfunctioning pituitary adenomas. Neurosurgery. 2016;79(4):E533–E535. 27635961 10.1227/NEU.0000000000001389

[16] Sheehan J , Lee CC, Bodach ME, et al Congress of neurological surgeons systematic review and evidence-based guideline for the management of patients with residual or recurrent nonfunctioning pituitary adenomas. Neurosurgery. 2016;79(4):E539–E540. 27635963 10.1227/NEU.0000000000001385

[17] Minniti G , Clarke E, Scaringi C, Enrici RM. Stereotactic radiotherapy and radiosurgery for non-functioning and secreting pituitary adenomas. Rep Pract Oncol Radiother. 2016;21(4):370–378. 27330422 10.1016/j.rpor.2014.09.004 PMC4899479

[18] Park KJ , Kano H, Parry PV, et al Long-term outcomes after Gamma Knife stereotactic radiosurgery for nonfunctional pituitary adenomas. Neurosurgery. 2011;69(6):1188–1199. 21552167 10.1227/NEU.0b013e318222afed

[19] Runge MJR , Maarouf M, Hunsche S, et al LINAC-radiosurgery for nonsecreting pituitary adenomas. Long-term results. Strahlenther Onkol. 2012;188(4):319–325. 22349709 10.1007/s00066-011-0052-5

[20] Hamblin R , Vardon A, Akpalu J, et al Risk of second brain tumour after radiotherapy for pituitary adenoma or craniopharyngioma: a retrospective, multicentre, cohort study of 3679 patients with long-term imaging surveillance. Lancet Diabetes Endocrinol. 2022;10(8):581–588. 35780804 10.1016/S2213-8587(22)00160-7

[21] Losa M , Spatola G, Albano L, et al Frequency, pattern, and outcome of recurrences after Gamma Knife radiosurgery for pituitary adenomas. Endocrine. 2017;56(3):595–602. 27688011 10.1007/s12020-016-1081-8

[22] Verma J , McCutcheon IE, Waguespack SG, Mahajan A. Feasibility and outcome of re-irradiation in the treatment of multiply recurrent pituitary adenomas. Pituitary. 2014;17(6):539–545. 24272035 10.1007/s11102-013-0541-x

[23] Breen P , Flickinger JC, Kondziolka D, Martinez AJ. Radiotherapy for nonfunctional pituitary adenoma: analysis of long-term tumor control. J Neurosurg. 1998;89(6):933–938. 9833818 10.3171/jns.1998.89.6.0933

[24] Brada M , Ashley S, Ford D, et al Cerebrovascular mortality in patients with pituitary adenoma. Clin Endocrinol (Oxf). 2002;57(6):713–717. 12460319 10.1046/j.1365-2265.2002.01570.x

[25] Sheehan JP , Starke RM, Mathieu D, et al Gamma Knife radiosurgery for the management of nonfunctioning pituitary adenomas: a multicenter study: clinical article. J Neurosurg. 2013;119(2):446–456. 23621595 10.3171/2013.3.JNS12766

[26] Sun S , Liu A, Zhang Y. Long-term follow-up studies of Gamma Knife radiosurgery for postsurgical nonfunctioning pituitary adenomas. World Neurosurg. 2019;124:e715–e723. 30660894 10.1016/j.wneu.2019.01.009

[27] Deng Y , Li Y, Li X, et al Long-term results of Gamma Knife Radiosurgery for Postsurgical residual or recurrent nonfunctioning Pituitary Adenomas. Int J Med Sci. 2020;17(11):1532–1540. 32669956 10.7150/ijms.47168 PMC7359386

[28] Dayawansa S , Abbas SO, Mantziaris G, et al Volumetric assessment of nonfunctional pituitary adenoma treated with stereotactic radiosurgery: an assessment of long-term response. Neurosurgery. 2022;93: https://doi.org/10.1227/neu.0000000000002594. 37437306

[29] Mantziaris G , Pikis S, Chytka T, et al Adjuvant versus on-progression Gamma Knife radiosurgery for residual nonfunctioning pituitary adenomas: a matched-cohort analysis. J Neurosurg. 2022;1(aop):1–7. 10.3171/2022.10.JNS221873 36401547

[30] Pomeraniec IJ , Xu Z, Lee CC, et al Dose to neuroanatomical structures surrounding pituitary adenomas and the effect of stereotactic radiosurgery on neuroendocrine function: an International multicenter study. J Neurosurg. 2022;136(3):813–821. 34560630 10.3171/2021.3.JNS203812

[31] Cordeiro D , Xu Z, Li CE, et al Gamma Knife radiosurgery for the treatment of Nelson’s syndrome: a multicenter, International study. J Neurosurg. 2019;133(2):336–341. 31299652 10.3171/2019.4.JNS19273

[32] Wolf A , Naylor K, Tam M, et al Risk of radiation-associated intracranial malignancy after stereotactic radiosurgery: a retrospective, multicentre, cohort study. Lancet Oncol. 2019;20(1):159–164. 30473468 10.1016/S1470-2045(18)30659-4

[33] El-Shehaby AMN , Reda WA, Tawadros SR, Abdel Karim KM. Low-dose Gamma Knife surgery for nonfunctioning pituitary adenomas. J Neurosurg. 2012;117(Suppl):84–88. 23205793 10.3171/2012.6.GKS12986

[34] Milano MT , Grimm J, Soltys SG, et al Single- and multi-fraction stereotactic radiosurgery dose tolerances of the optic pathways. Int J Radiat Oncol Biol Phys. 2021;110(1):87–99. 29534899 10.1016/j.ijrobp.2018.01.053 PMC9479557

[35] Cohen-Inbar O , Xu Z, Lee CC, et al Prognostic significance of corticotroph staining in radiosurgery for non-functioning pituitary adenomas: a multicenter study. J Neurooncol. 2017;135(1):67–74. 28913674 10.1007/s11060-017-2520-y PMC5817983

[36] Maragkos GA , Mantziaris G, Pikis S, et al Silent corticotroph staining pituitary neuroendocrine tumors: prognostic significance in radiosurgery. Neurosurgery. 2022;93: https://doi.org/10.1227/neu.0000000000002607. 37966247

[37] Brada M , Rajan B, Traish D, et al The long-term efficacy of conservative surgery and radiotherapy in the control of pituitary adenomas. Clin Endocrinol (Oxf). 1993;38(6):571–578. 8334743 10.1111/j.1365-2265.1993.tb02137.x

[38] Gittoes NJ , Bates AS, Tse W, et al Radiotherapy for non-function pituitary tumours. Clin Endocrinol (Oxf). 1998;48(3):331–337. 9578824 10.1046/j.1365-2265.1998.00393.x

[39] Minniti G , Scaringi C, Poggi M, et al Fractionated stereotactic radiotherapy for large and invasive non-functioning pituitary adenomas: long-term clinical outcomes and volumetric MRI assessment of tumor response. Eur J Endocrinol. 2015;172(4):433–441. 25627653 10.1530/EJE-14-0872

[40] Wilson PJ , De-Loyde KJ, Williams JR, Smee RI. A single centre’s experience of stereotactic radiosurgery and radiotherapy for non-functioning pituitary adenomas with the linear accelerator (Linac). J Clin Neurosci. 2012;19(3):370–374. 22277561 10.1016/j.jocn.2011.07.025

[41] Ronson BB , Schulte RW, Han KP, et al Fractionated proton beam irradiation of pituitary adenomas. Int J Radiat Oncol Biol Phys. 2006;64(2):425–434. 16257131 10.1016/j.ijrobp.2005.07.978

[42] Pomeraniec IJ , Dallapiazza RF, Xu Z, Jane JA, Sheehan JP. Early versus late Gamma Knife radiosurgery following transsphenoidal resection for nonfunctioning pituitary macroadenomas: a matched cohort study. J Neurosurg. 2016;125(1):202–212. 26517773 10.3171/2015.5.JNS15581

[43] Schoenthaler R , Albright NW, Wara WM, et al Re-irradiation of pituitary adenoma. Int J Radiat Oncol Biol Phys. 1992;24(2):307–314. 1526869 10.1016/0360-3016(92)90686-c

[44] Fleseriu M , Auchus R, Bancos I, et al Consensus on diagnosis and management of Cushing’s disease: a guideline update. Lancet Diabetes Endocrinol. 2021;9(12):847–875. 34687601 10.1016/S2213-8587(21)00235-7 PMC8743006

[45] Mehta GU , Ding D, Patibandla MR, et al Stereotactic radiosurgery for Cushing disease: results of an International, Multicenter Study. J Clin Endocrinol Metab. 2017;102(11):4284–4291. 28938462 10.1210/jc.2017-01385

[46] Devin JK , Allen GS, Cmelak AJ, Duggan DM, Blevins LS. The efficacy of linear accelerator radiosurgery in the management of patients with Cushing’s disease. Stereotact Funct Neurosurg. 2004;82(5–6):254–262. 15665560 10.1159/000083476

[47] Minniti G , Osti M, Jaffrain-Rea ML, et al Long-term follow-up results of postoperative radiation therapy for Cushing’s disease. J Neurooncol. 2007;84(1):79–84. 17356896 10.1007/s11060-007-9344-0

[48] Estrada J , Boronat M, Mielgo M, et al The long-term outcome of pituitary irradiation after unsuccessful transsphenoidal surgery in Cushing’s disease. N Engl J Med. 1997;336(3):172–177. 8988897 10.1056/NEJM199701163360303

[49] Petit JH , Biller BMK, Yock TI, et al Proton stereotactic radiotherapy for persistent adrenocorticotropin-producing adenomas. J Clin Endocrinol Metab. 2008;93(2):393–399. 18029460 10.1210/jc.2007-1220

[50] Loeffler JS , Shih HA. Radiation therapy in the management of pituitary adenomas. J Clin Endocrinol Metab. 2011;96(7):1992–2003. 21525155 10.1210/jc.2011-0251

[51] Wattson DA , Tanguturi SK, Spiegel DY, et al Outcomes of proton therapy for patients with functional pituitary adenomas. Int J Radiat Oncol Biol Phys. 2014;90(3):532–539. 25194666 10.1016/j.ijrobp.2014.06.068

[52] Papakokkinou E , Piasecka M, Carlsen HK, et al Prevalence of Nelson’s syndrome after bilateral adrenalectomy in patients with Cushing’s disease: a systematic review and meta-analysis. Pituitary. 2021;24(5):797–809. 34036460 10.1007/s11102-021-01158-z PMC8416875

[53] Reincke M , Albani A, Assie G, et al Corticotroph tumor progression after bilateral adrenalectomy (Nelson’s syndrome): systematic review and expert consensus recommendations. Eur J Endocrinol. 2021;184(3):P1–P16. 33444221 10.1530/EJE-20-1088 PMC8060870

[54] Bunevicius A , Lavezzo K, Smith PW, Vance ML, Sheehan J. Stereotactic radiosurgery before bilateral adrenalectomy is associated with lowered risk of Nelson’s syndrome in refractory Cushing’s disease patients. Acta Neurochir. 2021;163(7):1949–1956. 33759014 10.1007/s00701-021-04823-1

[55] Katznelson L , Laws ER, Jr, Melmed S, et al; Endocrine Society. Acromegaly: an endocrine society clinical practice guideline. J Clin Endocrinol Metab. 2014;99(11):3933–3951. 25356808 10.1210/jc.2014-2700

[56] Jenkins PJ , Bates P, Carson MN, Stewart PM, Wass JH. Conventional pituitary irradiation is effective in lowering serum growth hormone and insulin-like growth factor-I in patients with acromegaly. J Clin Endocrinol Metab. 2006;91(4):1239–1245. 16403824 10.1210/jc.2005-1616

[57] Knappe UJ , Petroff D, Quinkler M, et al; participants of the German Acromegaly Registry. Fractionated radiotherapy and radiosurgery in acromegaly: analysis of 352 patients from the German Acromegaly Registry. Eur J Endocrinol. 2020;182(3):275–284. 31917680 10.1530/EJE-19-0784

[58] Ding D , Mehta GU, Patibandla MR, et al Stereotactic radiosurgery for acromegaly: an International multicenter retrospective cohort study. Neurosurgery. 2019;84(3):717–725. 29757421 10.1093/neuros/nyy178 PMC6505445

[59] Kong DS , Kim YH, Kim YH, et al Long-term efficacy and tolerability of Gamma Knife radiosurgery for growth hormone-secreting adenoma: a retrospective multicenter study (MERGE-001). World Neurosurg. 2019;122:e1291–e1299. 30448582 10.1016/j.wneu.2018.11.038

[60] Mathieu D , Kotecha R, Sahgal A, et al Stereotactic radiosurgery for secretory pituitary adenomas: systematic review and International Stereotactic Radiosurgery Society practice recommendations. J Neurosurg. 2022;136(3):801–812. 34479203 10.3171/2021.2.JNS204440

[61] Melmed S , Bronstein MD, Chanson P, et al A Consensus Statement on acromegaly therapeutic outcomes. Nat Rev Endocrinol. 2018;14(9):552–561. 30050156 10.1038/s41574-018-0058-5 PMC7136157

[62] Melmed S , Casanueva FF, Hoffman AR, et al; Endocrine Society. Diagnosis and treatment of hyperprolactinemia: an endocrine society clinical practice guideline. J Clin Endocrinol Metab. 2011;96(2):273–288. 21296991 10.1210/jc.2010-1692

[63] Hung YC , Lee CC, Yang HC, et al The benefit and risk of stereotactic radiosurgery for prolactinomas: an international multicenter cohort study. J Neurosurg. 2019;1–10. 10.3171/2019.4.JNS183443 31374549

[64] Wan H , Chihiro O, Yuan S. MASEP Gamma Knife radiosurgery for secretory pituitary adenomas: experience in 347 consecutive cases. J Exp Clin Cancer Res. 2009;28(1):36. 19284583 10.1186/1756-9966-28-36 PMC2660297

[65] Pan L , Zhang N, Wang EM, et al Gamma knife radiosurgery as a primary treatment for prolactinomas. J Neurosurg. 2000;93(Suppl 3):10–13. 11143223 10.3171/jns.2000.93.supplement

[66] Tsang RW , Brierley JD, Panzarella T, et al Role of radiation therapy in clinical hormonally-active pituitary adenomas. Radiother Oncol. 1996;41(1):45–53. 8961367 10.1016/s0167-8140(96)91807-1

[67] Castinetti F , Nagai M, Dufour H, et al Gamma knife radiosurgery is a successful adjunctive treatment in Cushing’s disease. Eur J Endocrinol. 2007;156(1):91–98. 17218730 10.1530/eje.1.02323

[68] Pouratian N , Sheehan J, Jagannathan J, et al Gamma knife radiosurgery for medically and surgically refractory prolactinomas. Neurosurgery. 2006;59(2):255–66; discussion 255. 16883166 10.1227/01.NEU.0000223445.22938.BD

[69] Sala E , Moore JM, Amorin A, et al CyberKnife robotic radiosurgery in the multimodal management of acromegaly patients with invasive macroadenoma: a single center’s experience. J Neurooncol. 2018;138(2):291–298. 29429125 10.1007/s11060-018-2793-9

[70] Iwata H , Sato K, Nomura R, et al Long-term results of hypofractionated stereotactic radiotherapy with CyberKnife for growth hormone-secreting pituitary adenoma: evaluation by the Cortina consensus. J Neurooncol. 2016;128(2):267–275. 26961771 10.1007/s11060-016-2105-1

